# Niche-specific macrophage loss promotes skin capillary aging

**DOI:** 10.1101/2023.08.25.554832

**Published:** 2023-08-27

**Authors:** Kailin R. Mesa, Kevin A. O’Connor, Charles Ng, Steven P. Salvatore, Dan R. Littman

**Affiliations:** 1 Department of Cell Biology, New York University School of Medicine, New York, NY 10016, USA; 2 Department of Pathology and Laboratory Medicine, Weill Cornell Medicine, New York-Presbyterian Hospital, New York, NY 10021, USA; 3 Perlmutter Cancer Center, New York University Langone Health, New York, NY 10016, USA; 4 Howard Hughes Medical Institute, New York, NY 10016, USA

**Keywords:** Aging, Resident Macrophages, Blood Capillaries, Tissue Repair, Intravital Microscopy

## Abstract

All mammalian organs depend upon resident macrophage populations to coordinate repair processes and facilitate tissue-specific functions^1–3^. Recent work has established that functionally distinct macrophage populations reside in discrete tissue niches and are replenished through some combination of local proliferation and monocyte recruitment^4,5^. Moreover, decline in macrophage abundance and function in tissues has been shown to contribute to many age-associated pathologies, such as atherosclerosis, cancer, and neurodegeneration^6–8^. Despite these advances, the cellular mechanisms that coordinate macrophage organization and replenishment within an aging tissue niche remain largely unknown. Here we show that capillary-associated macrophages (CAMs) are selectively lost over time, which contributes to impaired vascular repair and tissue perfusion in older mice. To investigate resident macrophage behavior *in vivo*, we have employed intravital two-photon microscopy to non-invasively image in live mice the skin capillary plexus, a spatially well-defined model of niche aging that undergoes rarefication and functional decline with age. We find that CAMs are lost with age at a rate that outpaces that of capillary loss, leading to the progressive accumulation of capillary niches without an associated macrophage in both mice and humans. Phagocytic activity of CAMs was locally required to repair obstructed capillary blood flow, leaving macrophage-less niches selectively vulnerable to both homeostatic and injury-induced loss in blood flow. Our work demonstrates that homeostatic renewal of resident macrophages is not as finely tuned as has been previously suggested^9–11^. Specifically, we found that neighboring macrophages do not proliferate or reorganize sufficiently to maintain an optimal population across the skin capillary niche in the absence of additional cues from acute tissue damage or increased abundance of growth factors, such as colony stimulating factor 1 (CSF1). Such limitations in homeostatic renewal and organization of various niche-resident cell types are potentially early contributors to tissue aging, which may provide novel opportunities for future therapeutic interventions.

Tissue homeostasis is dependent on multiple macrophage populations that reside in distinct sub-tissue compartments or niches, such as epithelium, blood vessels or nerves, and are thought to support specialized tissue functions^4,12,13^. Recent work has suggested that functional decline and rarefication of vascular niches may contribute to various age-associated tissue pathologies (including sarcopenia, chonic wounds, and Alzheimer’s disease)^14–16^. It is not yet known how tissue-resident macrophages resist or potentiate such niche specific aging processes^6,17^.

## Capillary-associated macrophage decline with age correlates with impaired blood flow.

**Extended Data Figure 1.**
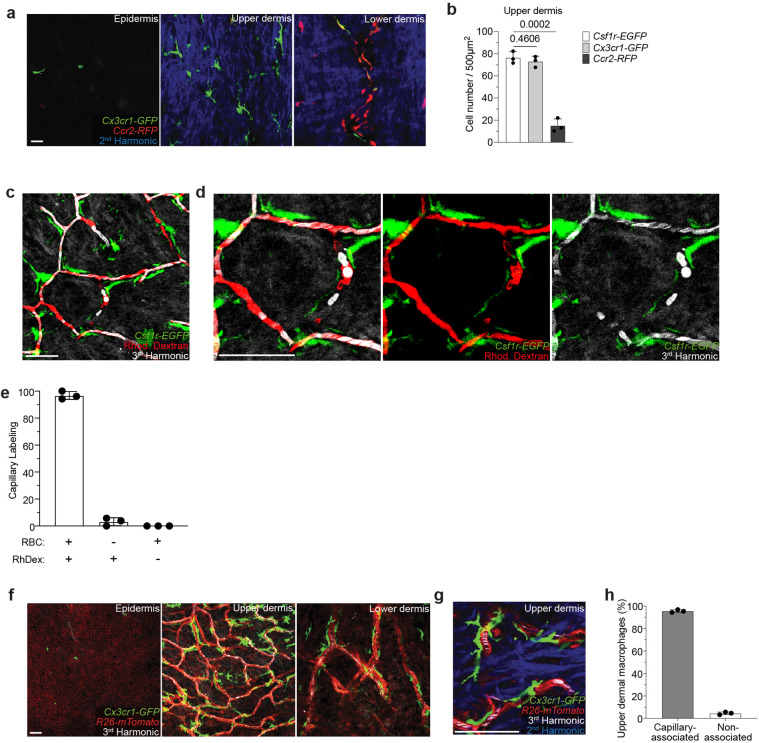
CX3C-chemokine receptor 1 (CX3CR1) expression in the upper dermis defines a resident population of capillary-associated macrophages (CAMs). a) Representative images of cells expressing *Ccr2-RFP*, *Csf1r-EGFP*, or *Cx3cr1-GFP* in the epidermis, upper dermis, and lower dermis of 1 month old mice. b) Quantifications of cells expressing *Csf1r-EGFP*, *Cx3cr1-GFP, or Ccr2-RFP*, in the upper dermis (n = 3 mice in each group; two 500μm^2^ regions per mouse; Cell number (*Csf1r-EGFP* vs *Cx3cr1-GFP; Csf1r-EGFP* vs *Ccr2-RFP*) was compared by unpaired Student’s t test; mean ± SD). c-d) Simultaneous visualization of blood flow through the superficial capillary plexus via third harmonic generation (white) from red blood cells (RBC) and intravenous rhodamine dextran (RhDex) (red). e) Quantifications of RBC and RhDex labeling of the upper dermal superficial capillary plexus (n = 3 mice in total; four 500μm^2^ regions per mouse; mean ± SD). f) Visualization of CX3CR1-expressing cells in all skin layers: epidermis, upper dermis, and lower dermis (*Cx3cr1-GFP;R26-mTmG*) was performed during homeostatic conditions. g) Representative image of labeled upper dermal macrophages in contact with capillary superficial plexus (red) in 1 month old mice. h) Quantifications reveal most upper dermal macrophages are capillary-associated macrophages (CAMs) (n = 3 mice in total; three 500μm^2^ regions per mouse; mean ± SD) . Scale bar, 50 μm.

**Extended Data Figure 2.**
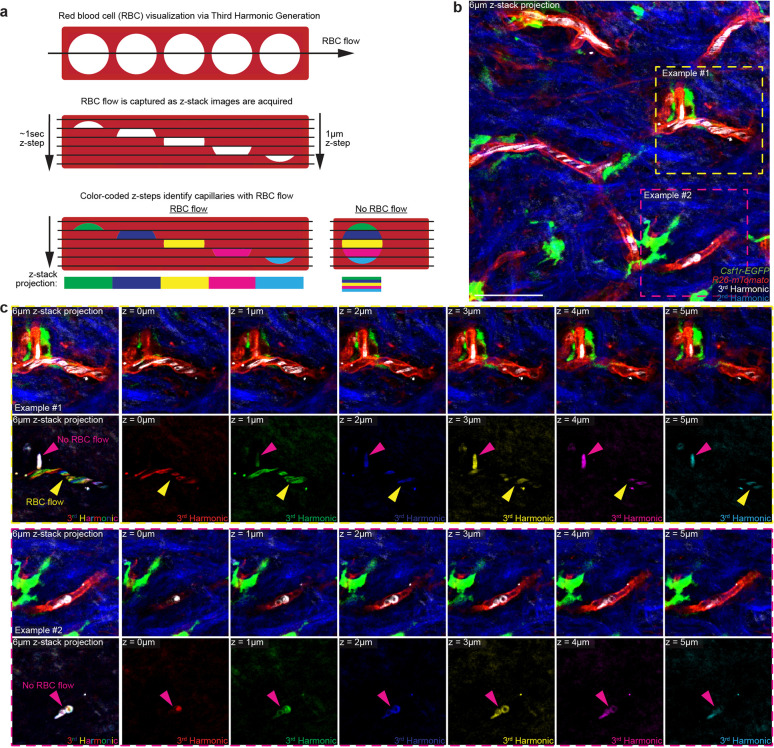
Label-free *in vivo* visualization of capillary blood flow via third harmonic effect generated by red blood cells. **a,** Scheme of red blood cell (RBC) flow through a segment of the superficial skin capillary network. During three-dimensional image acquisition, flowing red blood cells are captured at different x,y positions for each z-section along the capillary segment. Pseudocoloring each z-step through a capillary segment distinguishes flowing RBCs as a multicolor patchwork or rainbow-effect and leaves obstructed/non-flowing RBCs as white (full overlap of all colors). **b,** Representative image of RBC visualization in the upper dermis in *Csf1r-EGFP; R26-mTmG* mice. **c,** Representative optical z-sections through upper dermal capillaries. Third harmonic signal is pseudocolored differently for each z-step to visualize RBC movement along the capillary segments during image acquisition. Scale 50μm.

**Extended Data Figure 3.**
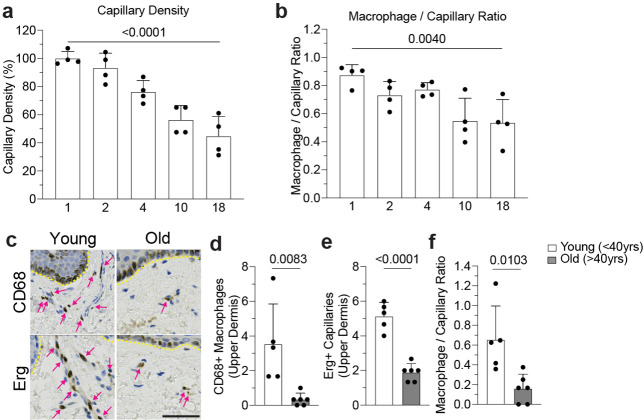
Capillary and associated macrophage loss in mice and humans. **a,b** Quantification of capillary niche age-associated changes, (a) Capillary density and (b) CAM / Capillary segment ratio (n = 4 mice in each age group; two 500μm^2^ regions per mouse; comparison across age groups was by one-way ANOVA; mean ± SD). **c,** Representative immunohistochemistry of CD68+ macrophage and Erg+ capillary density in the upper dermis of both young (<40y) and old (>40y) human samples. **d-f,** Quantification of capillary niche age-associated changes: (d) CD68+ macrophage density, (e) Erg+ capillary density, (f) Upper dermal macrophage / Capillary endothelium ratio (n = 5 patient samples in each group; three imaging regions per sample; Comparison by unpaired Student’s t test; mean ± SD). Scale bar, 50μm.

**Extended Data Figure 4.**
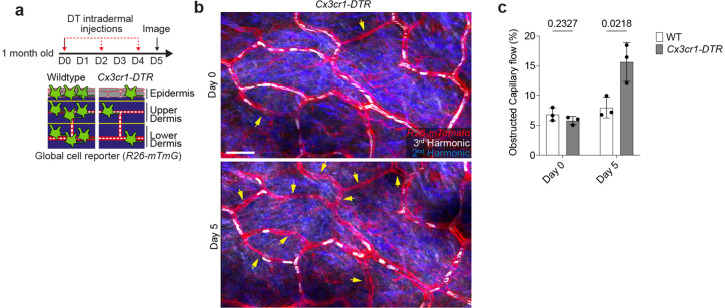
Impaired skin capillary blood flow following acute macrophage depletion. **a,** Scheme of long-term capillary blood flow tracking following intraperitoneal injection every other day with diphtheria toxin (DT) (25ng/g body weight in PBS) in both *Cx3cr1-DTR*; *R26-mTmG* and WT control (*R26-mTmG*) mice. **b,** Representative revisits of the same capillary network to visualize capillaries (*R26-mTmG*) and RBC flow (Third Harmonic). **c,** Quantifications reveal a significant reduction in the percentage of capillaries with blood flow following DT-induced cell depletion (n = 647 capillary segments in WT control regions, n = 695 capillary segments in *Cx3cr1-DTR* regions; n = 3 mice in each group; obstructed capillary flow (WT vs *Cx3cr1-DTR*) was compared for Day 0 and Day 5 time points by unpaired Student’s t test; mean ± SD). Scale bar, 50 μm.

**Figure 1 –:**
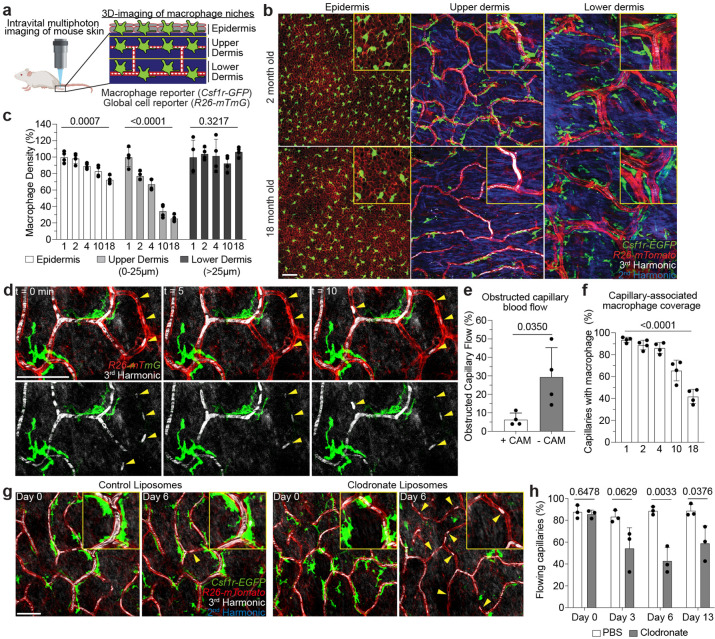
Niche-specific macrophage loss with age correlates with impaired skin capillary blood flow. **a,** Schematic of intravital imaging of resident macrophage populations in mouse skin, indicating epidermal and dermal populations, using the macrophage reporter, *Csf1r-EGFP,* in combination with an *Actb*-driven universal tdTomato reporter, *R26-mTmG*. **b,** Representative optical sections of distinct resident macrophage populations in young (2-month-old) and old (18-month-old) mice. **c,** Quantification of niche-specific macrophage density change in 1, 2, 4, 10, and 18 month old mice (n = 4 mice in each age group; two 500μm^2^ regions per mouse; macrophage density in each skin niche was compared across age groups by one-way ANOVA; mean ± SD). **d,** Skin resident macrophage labeling using *Cx3cr1-CreERT2;R26-mTmG* mice was performed following a single high-dose intraperitoneal injection of tamoxifen (2mg) in 1 month old mice. Single optical sections at successive time points 5 min apart showing red blood cell (RBC) flow (white) in capillaries (red) with or without nearby CAMs (green). Yellow arrowheads indicate obstructed RBC capillary flow. **e,** Quantification of obstructed capillary flow as measured by stalled RBCs as described in Extended Data Figure 2 (n = 226 CAM+ capillary segments, n = 27 CAM− capillary segments; n = 4 mice; obstructed capillary flow (CAM+ vs CAM−) was compared by paired Student’s t test; mean ± SD). **f,** Percentage of capillary segments with at least one associated macrophage (n = 4 mice in each age group; two 500μm^2^ regions per mouse; macrophage association with capillary segments was compared across age groups by one-way ANOVA; mean ± SD). **g,** Representative images demonstrate macrophage depletion following intradermal injections of clodronate-liposomes every 3 days. Repeated intravital imaging of the vascular niche was performed to visualize macrophages (*Csf1r-GFP*), capillaries (*R26-mTmG*) and RBC flow (Third Harmonic). **h,** Percentage of capillaries with blood flow following macrophage depletion (n = 194 capillary segments in clodronate group, n = 199 capillary segments in PBS group; 3 mice in each group; capillary flow (clodronate vs PBS) was compared by unpaired Student’s t test; mean ± SD). Scale bar, 50 μm.

To model mammalian tissue aging, we adapted an intravital microscopy technique to visualize skin resident macrophage populations non-invasively in live mice throughout the lifetime of the organism^18,19^ (Figure 1a, Supplemental Videos 1 and 2). Unexpectedly, longitudinal imaging of skin macrophages (marked by *Csf1r-EGFP*) revealed niche-specific decline in macrophage populations. Subdividing the skin into three anatomical layers, epidermis, upper (papillary) and lower (reticulated) dermis, we observed that macrophages of the upper dermis were lost with age at a greater rate than macrophages from both epidermis and lower dermis (Figure 1b–c). Further characterization of this population with additional myeloid fluorescent reporters revealed that most macrophages of the upper dermis express the chemokine receptor CX_3_CR1, but not CCR2 (Extended Data Fig. 1a,b). A major component of the upper dermal niche is the superficial capillary plexus, which supplies nutrient exchange for the overlying epidermis. To visualize this structure, we utilized third harmonic generation from our imaging to track red blood cell (RBC) flow^20,21^, which was consistent with conventional labeling methods such as rhodamine dextran injection (Extended Data Fig. 1c–e and Supplemental Video 3). With this *in vivo* marker of blood flow, we found that the macrophages were closely associated with blood capillaries of the superficial plexus, suggesting that they may provide support for this capillary niche (Extended Data Fig. 1f–h and Supplemental Video 4). To investigate if these macrophages play a role in capillary function, we assessed if blood flow, via RBC flow (Extended Data Fig. 2), was altered in the presence of capillary-associated macrophages (CAMs). Performing timelapse recordings of fluorescently labeled CAMs in mice with a cre-dependent dual reporter system (*Cx3cr1-CreERT2; R26-mTmG*), we found that capillaries lacking an associated macrophage had a higher rate of obstructed blood flow (Figure 1d,e and Supplemental Video 5). Longitudinal imaging across multiple ages showed that the fraction of capillaries with CAMs significantly decreased with age (Figure 1f). This decrease in CAMs and coverage outpaced the loss of capillaries (Extended Data Fig. 3a,b), which was previously shown to be an early hallmark of aging in multiple tissues, including central nervous system, lung, kidney, and skin^14,22–26^. To assess if this phenomenon also occurs in humans, we obtained both young and old human patient skin samples. Consistent with our observations in mice, human capillary-associated macrophages also displayed a decline with age. Moreover, CAM decline also outpaced capillary loss with age, suggesting a similar loss in macrophage coverage of the capillary niche (Extended Data Fig. 3c–f). To assess any functional role of CAMs in maintaining capillary blood flow, we performed chemical and genetic ablation of CAMs, via clodronate liposomes and *Cx3cr1-DTR* depletion, respectively, and observed an acute loss in capillary flow (Figure 1 g,h and Extended Data Fig. 4). Collectively, this work highlights an evolutionarily conserved loss in skin capillary-associated macrophages with age, which correlates with impaired homeostatic capillary perfusion.

## Local CAM recruitment and phagocytosis is required to restore blood flow.

**Extended Data Figure 5.**
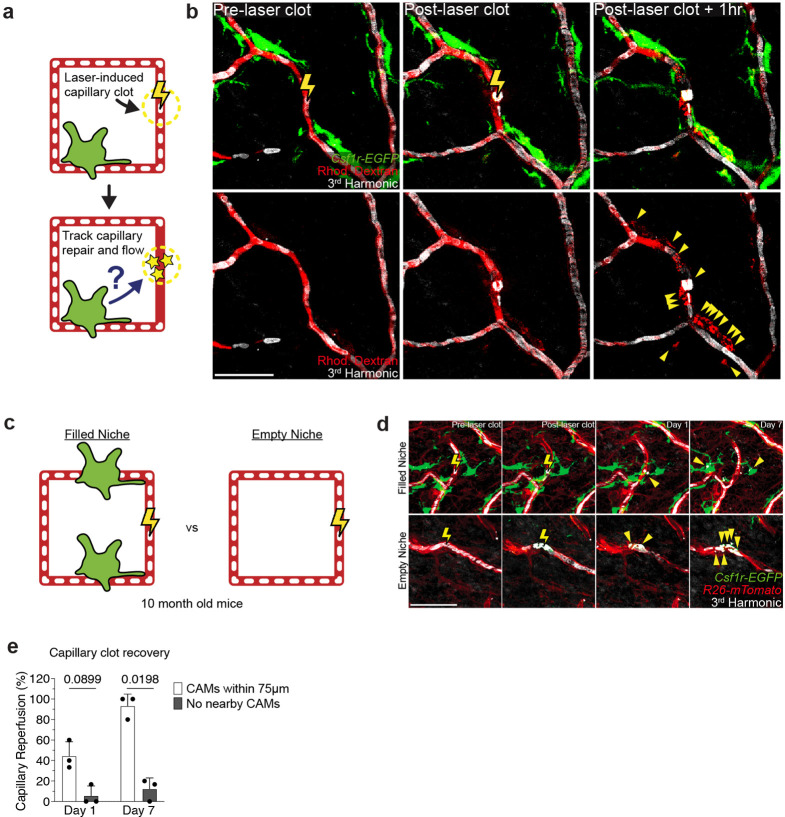
Laser-induced model of acute capillary clot formation and repair. **a,** Scheme of laser-induced capillary clot experiment. **b,** Sequential revisits of damaged capillary niche after laser-induced clot formation in *Csf1r-EGFP* mice. Simultaneous visualization of blood flow before and after clot formation via third harmonic generation (white) from red blood cells (RBC) and intravenous rhodamine dextran (RhDex) (red). Yellow lightning bolt indicates site of laser-induced capillary clot. Yellow arrowheads indicate extra-luminal vascular debris. **c,** Scheme of laser-induced capillary clot in niches with or without CAMs. **d,** Sequential revisits of damaged capillary niche after laser-induced clot formation in *Csf1r-EGFP;R26-mTmG* mice. Capillary clot formation (yellow lightning bolt) was performed at 940nm for 1s in 10 month old mice. Yellow arrowheads indicate extra-luminal vascular debris. **e,** Quantification of capillary reperfusion at day 1 and 7 after laser-induced clotting (n = 16 capillary clots in regions with CAMs (<75μm from clot), n = 16 capillary clots in regions without CAMs (>75μm from clot; 3 mice in total; capillary reperfusion (with CAMs vs without CAMs) was compared at Day 1 and Day 7 by paired Student’s t test; mean ± SD). Scale bar, 50 μm.

**Figure 2 –:**
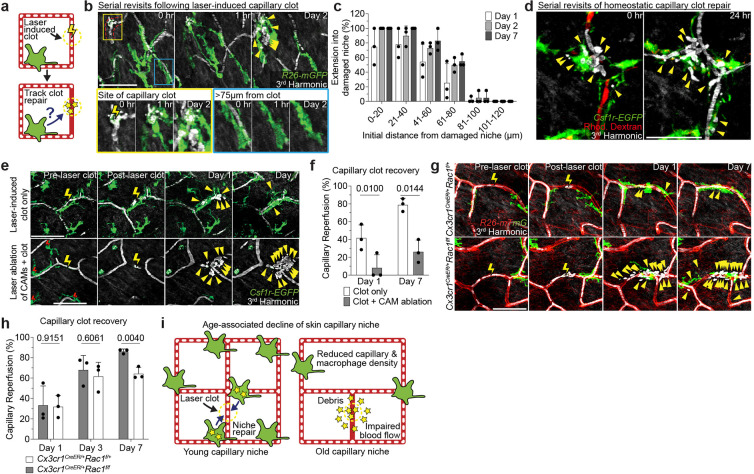
Local CAM recruitment and phagocytosis are required to restore impaired blood flow. **a,** Scheme of laser-induced capillary clot experiment. **b,** Sequential revisits of damaged capillary segment (red dashed lines) after laser-induced clot formation in *Cx3cr1-CreERT2;R26-mTmG* mice. Yellow lightning bolt indicates site of laser-induced capillary clot. Yellow arrowheads indicate extra-luminal vascular debris. **c,** Quantification of capillary-associated macrophage extension toward damaged niche at Day 1, 2 and 7 after laser-induced clotting (n = 50 CAMs, in 3 mice; mean ± SD). **d,** Sequential revisits of naturally forming capillary clots in *Csf1r-EGFP* mice. Simultaneous visualization of blood flow during clot repair via third harmonic generation (white) from red blood cells (RBC) and intravenous rhodamine dextran (RhDex) (red). **e,** Sequential revisits of damaged capillary niche after laser-induced CAM ablation and clot formation in *Csf1r-EGFP* mice. Macrophage laser ablation (red lightning bolt) and capillary clot formation (yellow lightning bolt) were both performed at 940nm for 1s. **f,** Quantification of capillary reperfusion at Day 1 and 7 after laser-induced clotting and macrophage ablation (n = 19 capillary clots in CAM ablated regions, n = 16 capillary clots in control regions; 3 mice in total; capillary reperfusion (CAM ablated vs control) was compared by paired Student’s t test; mean ± SD). **g,** Sequential revisits of damaged capillary niche after laser-induced clot formation in *Cx3cr1*^*CreER*^;*Rac1*^*fl/fl*^ and *Cx3cr1*^*CreER*^;*Rac1*^*fl/+*^ mice. CAMs (green), capillaries (red), RBC (white). **h,** Quantification of capillary reperfusion at Day 1, 3 and 7 after laser-induced clotting (n = 67 clots in *Rac1*^*fl/+*^ group; n = 82 clots in *Rac1*^*fl/fl*^ group; n = 3 mice in each group; capillary reperfusion (*Rac1*^*fl/+*^ vs *Rac1*^*fl/fl*^) was compared by unpaired Student’s t test; mean ± SD). **i,** Scheme of age-associated decline of skin capillary niche. Capillary-associated macrophage density declines with age, which predisposes aged capillaries to impaired repair and sustained tissue perfusion. Scale bar, 50μm.

We next aimed to assess the cellular mechanism(s) by which CAMs support capillary blood flow, using a laser-induced blood clotting model to precisely target and stop blood flow in individual capillary segments (Figure 2a, Extended Data Fig. 5a,b and Supplemental Video 6). To assess any macrophage involvement, we tracked the daily displacement of surrounding CAMs to laser-induced clots. These data showed that CAM recruitment to sites of capillary damage as well as RBC engulfment are locally restricted to approximately 80–100μm, and largely occur within the first two days after injury (Figure 2b,c). Under homeostatic conditions, neighboring CAMs were similarly involved locally, taking up RBC debris from naturally occurring capillary clots (Figure 2d).

Given these findings, we next tested if capillary blood flow was preferentially repaired when at least one resident CAM was in close proximity (<75μm) with the injured capillary segment. The results showed a significant improvement in capillary repair for vessels with an associated macrophage (Extended Data Fig. 5c–e), suggesting that the loss of CAMs could lead to compromised capillary blood flow with age. To rule out an indirect association of capillary repair and CAM proximity, we performed laser-induced ablation of CAMs adjacent to capillary segments immediately prior to inducing capillary clots. Indeed, in regions with CAM ablation within 75μm of the capillary clot, repair was significantly impaired and blood flow was not properly reestablished (Figure 2e,f).

**Extended Data Figure 6.**
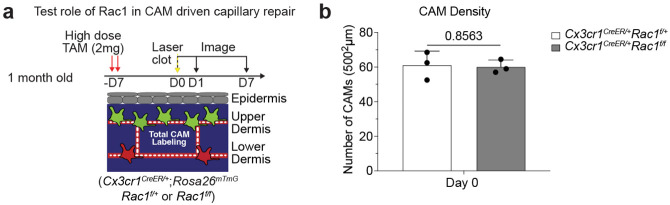
CAM density in mice with deficiencies in macrophage phagocytic machinery. **a,** Scheme of laser-induced capillary clot in *Cx3cr1*^*CreER*^;*Rac1*^*fl/fl*^ and *Cx3cr1*^*CreER*^;*Rac1*^*fl/+*^ mice. **b,** Quantification of CAM density at Day 7 after laser-induced clotting (n = 3 mice in each group; two 500μm^2^ regions per mouse; CAM density (*Rac1*^*fl/+*^ vs *Rac1*^*fl/fl*^) was compared at day 0 (same day of clot induction) by unpaired Student’s t test; mean ± SD).

Our serial imaging revealed that CAMs at capillary repair sites often contained RBC debris shortly after clot formation. To understand if CAM uptake and clearance of this cellular debris is functionally important for capillary repair, we acutely impaired CAM phagocytosis through inducible cre-dependent knockout of Rac1, a critical component of the phagocytic machinery^27^, one week prior to laser-induced clot formation. While there was no significant change in CAM density (Extended Data Fig. 6a,b), we found significant impairment in capillary repair in *Cx3cr1*^*CreER*^;*Rac1*^*fl/fl*^ mice compared to *Cx3cr1*^*CreER*^;*Rac1*^*fl/+*^ littermate controls (Figure 2g,h), suggesting that Rac1-dependent phagocytic clearance is critical for proper capillary repair and tissue reperfusion. Together, our results support a model in which local CAM recruitment and phagocytic clearance of capillary debris is critical to maintain capillary function. Therefore, as CAM density declines with age, so does capillary perfusion of the tissue (Figure 2i).

## CAMs do not replenish lost neighbors, resulting in population loss with age

**Extended Data Figure 7.**
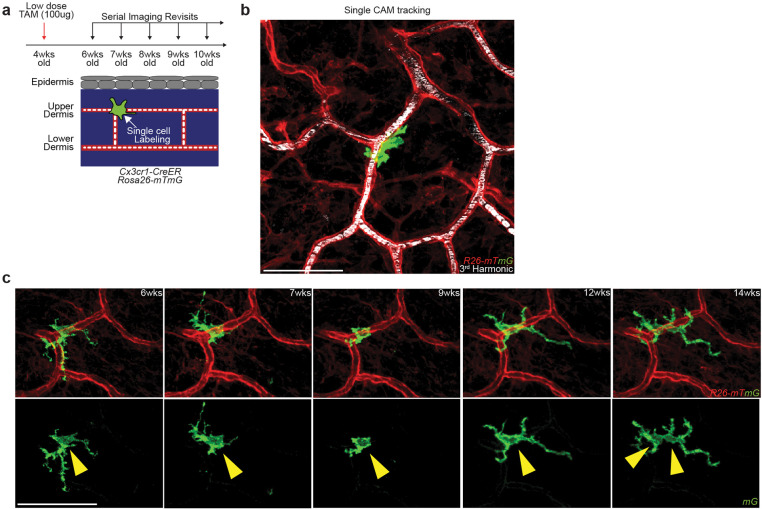
Single-cell *in vivo* lineage tracing of resident capillary-asssociated macrophages. **a,** Scheme of long-term tracking of CAM self-renewal experiment. **b,** Representative image of single macrophage tracing in *Cx3cr1-CreERT2*; *R26-mTmG* mice. **c,** Representative serial revisit images of single macrophage tracing in *Cx3cr1-CreERT2*; *R26-mTmG* mice.

**Figure 3 –:**
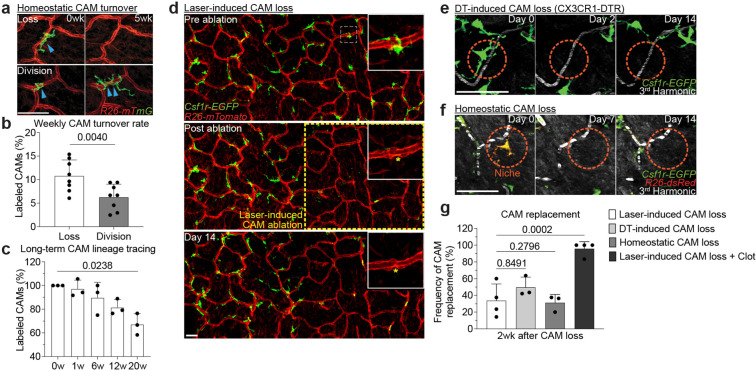
Capillary-associated macrophages do not replenish lost neighbors, resulting in population loss with age. **a,** Representative revisits of single macrophage lineage tracing (blue arrowhead) in *Cx3cr1-CreERT2*; *R26-mTmG* mice. Weekly revisits were performed during homeostatic conditions for 20 weeks following a single low-dose intraperitoneal injection of tamoxifen (50μg). **b,c,** Quantification of (b) weekly rate of CAM loss and division (n = 262 CAMs; 8 mice in total; CAM turnover rates (CAM loss vs division) was compared by paired Student’s t test; mean ± SD) and of (c) maintenance of the labeled CAM population over 20wk lineage tracing (n = 59 CAMs; 3 mice in total; number of fluorescently-labeled CAMs was compared across each time point in the same mice by one-way repeated measures ANOVA with Geisser-Greenhouse correction; mean ± SD ). **d,** Representative revisits of CAM replacement after laser-induced CAM ablation in *Csf1r-EGFP;R26-mTmG* mice. Macrophage laser ablations (yellow asterisk) were performed in a 500μm^2^ region (yellow dashed line square), **e,** Representative revisits of CAM replacement after a single intraperitoneal injection diphtheria toxin (10ng/g body weight in PBS) in *Cx3cr1-DTR* mice. **f,** Representative revisits of single lineage-tracked macrophages in *Cx3cr1-CreERT2*; *R26-dsRed*; *Csf1r-EGFP* mice. Weekly revisits were performed during homeostatic conditions for 2 weeks following a single low-dose intraperitoneal injection of tamoxifen (50μg). **g,** Quantification of replenishment rate of empty CAM niche under laser-induced loss (n = 44 CAMs; 4 mice in total), DT-induced loss (n = 53 CAMs; 3 mice in total), homeostatic loss (n = 29 CAMs; 3 mice in total), or laser-induced CAM loss with capillary clot (n = 25 CAMs; 4 mice in total). Frequency of CAM replacement after forced and homeostatic CAM loss were compared by unpaired Student’s t test; mean ± SD. Scale bar, 50μm.

Maintenance of tissue resident macrophage populations in the skin is thought to be mediated through a combination of local proliferation and systemic replacement by blood monocytes^13,28,29^. To understand how CAM density declines with age, we performed single-macrophage lineage tracing, by inducing sparse cre-recombination to label and tracking of individual macrophages over weekly revisits (Extended Data Fig. 7a–c). In doing so, we observed discrete behaviors, including macrophage loss and proliferation (Figure 3a). Analysis of weekly rate of CAM loss and division revealed a significant skew toward more CAM loss than division in the first 4 months of age (Figure 3b). Consistent with these data, there was a significant decline in the fraction of fate-mapped CAMs over a 20-week time course (Figure 3c). These results demonstrate that the CAMs are insufficiently replenished by local proliferation, which contributes to their progressive decline in this tissue niche.

**Extended Data Figure 8.**
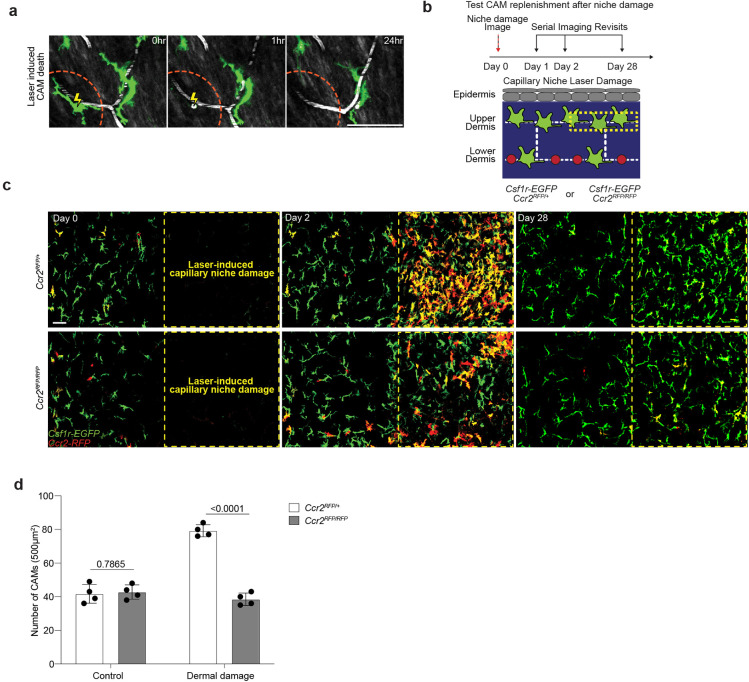
Injury-induced replenishment of CAM population is partially CCR2-dependent. **a,** Representative revisits of CAM replacement after laser-induced CAM ablation in *Csf1r-EGFP* mice. Macrophage laser ablations (yellow lightning bolt) were performed to leave an empty capillary niche (orange dashed line). **b,** Scheme of CAM replacement after capillary niche damage in *Csf1r-EGFP*; *Ccr2*^*RFP/+*^ or *Ccr2*^*RFP/RFP*^ mice. **c,** Representative revisits of CAM replacement after a regional laser-induced damage to the capillary niche. **d,** Quantification of replenishment rate (*Csf1r-EGFP*+ cells) of empty CAM niche after laser-induced CAM loss and niche damage (n = 4 mice per group; two 500μm^2^ regions per mouse; CAM density (*Ccr2*^*RFP/+*^ vs *Ccr2*^*RFP/RFP*^) was compared by unpaired Student’s t test; mean ± SD). Scale bar, 50μm.

The ability of resident macrophages to locally self-renew has largely been studied through methods of near-total macrophage depletion, which have limited niche-specificity and often generate tissue-wide inflammation^9–11^. To directly interrogate the steps of macrophage self-renewal over time, we tracked both the replacement of individual macrophages after loss and the distribution of sister macrophages after division. First, to track local macrophage replacement after CAM loss, we performed laser-induced ablation of all CAMs within a defined 500μm^2^ region. Serial revisits up to two weeks after ablation revealed minimal repopulation from adjacent capillary regions that retained intact CAM populations (Extended Data Fig. 8a and Figure 3d). We found a similar lack in repopulation following partial CAM depletion in *Cx3cr1*^*DTR*^ mice following low dose diphtheria toxin administration (Figure 3e). To avoid any non-physiological effects from these cell depletion models, we developed a dual fluorescent macrophage reporter mouse, *Cx3cr1-CreERT2; Rosa26-dsRed; Csf1r-EGFP*, allowing for cre-dependent recombination to differentially label a small fraction of macrophages and track their homeostatic replacement. Examination on serial weekly revisits indicated that, as we observed in the depletion models, most capillary niches did not recruit a new macrophage for as long as two weeks following CAM loss (Figure 3f). In contrast, emptied capillary niches were readily replenished with new CAMs when laser-induced macrophage loss was accompanied by laser-induced capillary damage (Figure 3g). Such replenishment was partially CCR2-dependent, suggestive that monocytes may participate in repopulating the capillary niche after injury (Extended Data Fig. 8b–d and Figure 3g). Taken together, these results suggest that CAM loss is not a sufficient trigger to promote neighboring macrophages into the emptied niche.

**Extended Data Figure 9.**
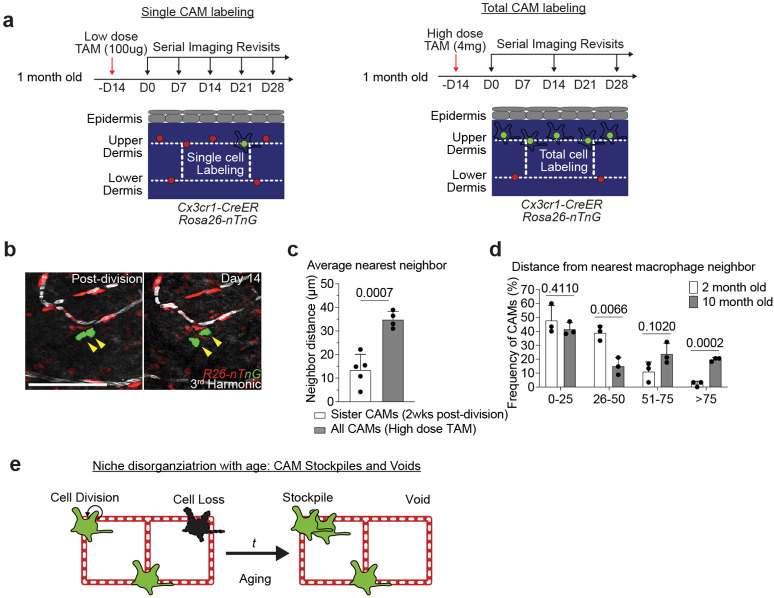
Spatially uncoupled proliferation and loss progressively lead to disorganized macrophage niche patterning. **a,** Scheme of long-term tracking of CAM migration following cell division. **b,** Representative revisits of single macrophage lineage tracing in *Cx3cr1-CreERT2*; *R26-nTnG* mice. Weekly revisits were performed during homeostatic conditions for 5 weeks following a single low-dose intraperitoneal injection of tamoxifen (50μg). **c,** Quantification of neighboring CAM distance of recently divided sister CAMs was compared to total CAM neighboring distance from *Cx3cr1-CreERT2*; *R26-nTnG* mice given a single high-dose intraperitoneal injection of tamoxifen (4mg) (n = 26 sister CAM pairs, from 5 mice in low-dose group; n = 188 CAMs, from 4 mice in high-dose group; Average nearest neighbor distance (2 vs 10 month old) was compared at 0–25, 26–50, 51–75, and >75μm intervals by unpaired Student’s t test mean ± SD). **d,** Quantification of distance between nearest CAM neighbors in 2 and 10 month old *Csf1r-EGFP* mice (n = 90 CAMs in 2 month old mice, n = 101 CAMs in 10 month old mice; 3 mice in each age group; Frequency of CAM distribution (2 vs 10 month old) was compared at 0–25, 26–50, 51–75, and >75μm intervals by unpaired Student’s t test; mean ± SD). **e,** Working model of macrophage renewal and organization in the skin aging capillary niche. Scale bar, 50μm.

Second, to precisely track sister macrophage migration following cell division, we studied mice with a dual fluorescent nuclear reporter, *Cx3cr1-CreERT2; R26-nTnG*, where we can use low or high doses of tamoxifen to either label individual or all CAMs, respectively (Extended Data Fig. 9a). Compared to the average distance between all neighboring macrophages, sister CAMs remained significantly closer to each other even 2 weeks after division (Extended Data Fig. 9b,c). These findings further support the notion that CAM division and loss are not spatiotemporally coupled, which we predict would progressively lead to disorganized patterning and the accumulation of both empty and crowded capillary niches. We tested this prediction by looking at the distribution of neighboring CAMs in both young (2-month old) and old (10-month old) mice. In young mice, the majority of macrophages were within 50μm of each other. In contrast, old mice had a biphasic distribution of macrophage patterning, with most CAMs either within 25μm or further than 75μm apart (Extended Data Fig. 9d). These results highlight two distinct cellular features that contribute to reduced CAM coverage with age: 1) insufficient macrophage repopulation following CAM loss, and 2) insufficient distribution of these cells along the capillary niche, which may promote progressive erosion of the vascular bed (Extended Data Fig. 9e).

## Local CAM replenishment in old mice is sufficient to rejuvenate capillary repair and tissue reperfusion

**Extended Data Figure 10.**
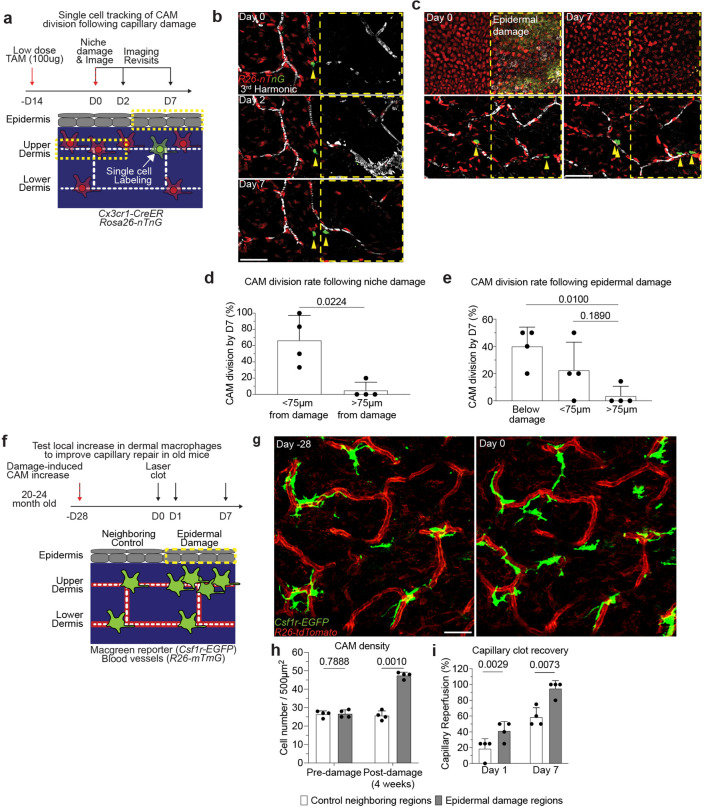
Large niche damage expands CAM population and improves capillary repair in old mice. **a,** Scheme of tracking of CAM proliferation following laser-induced damage to nearby capillary or epidermal niches. **b,c,** Representative revisits of single macrophage lineage tracing in *Cx3cr1-CreERT2*; *R26-nTnG* mice following (b) capillary and (c) epidermal damage. **d,e,** Quantification of CAM proliferation based on proximity to (d) capillary and (e) epidermal damage (n = 25 CAMs tracked in capillary damage regions, n = 56 CAMs tracked in epidermal damage regions; 4 mice in each group; CAM proliferation (based on damage proximity) was compared at day 7 by paired Student’s t test; mean ± SD). **f,** Scheme of local damage-induced expansion of CAMs in the aged capillary niche. **g,** Representative images of CAM density in *Csf1r-EGFP; R26-mTmG* mice 28 days following overlaying epidermal laser damage. **h,** Quantification of CAM density following overlaying epidermal damage (n = 4 mice in total; two 500μm^2^ regions for both epidermal damage and control conditions per mouse; CAM density (Epidermal damage vs Control) was compared by paired Student’s t test; mean ± SD). **i,** Quantification of capillary reperfusion at day 1 and 7 after laser-induced clotting (n = 17 capillary clots in epidermal damage regions, n = 17 capillary clots in neighboring control regions; 4 mice in total; capillary reperfusion (with epidermal damage vs control) was compared at day 1 and day 7 by paired Student’s t test; mean ± SD). following laser-induced damage to overlaying epidermal niche. Scale bar, 50μm.

While CAM proliferation was insufficient for maintenance of the population during homeostasis, we asked if extrinsic cues such as broader tissue damage could enhance division rates. We employed large laser-induced wounds in both the epidermal and upper dermal niches (Extended Data Fig. 10a) and found that local CAM proliferation was significantly increased one week following tissue damage in both layers (Extended Data Fig. 10b–e). Given these results we hypothesized that damage-induced CAM proliferation in the aged capillary niche would provide lasting increases in macrophage density. Indeed, broad epidermal damage resulted in a lasting increase in CAMs in damaged regions compared to neighboring control regions (Extended Data Fig. 10f,h).

**Figure 4 –:**
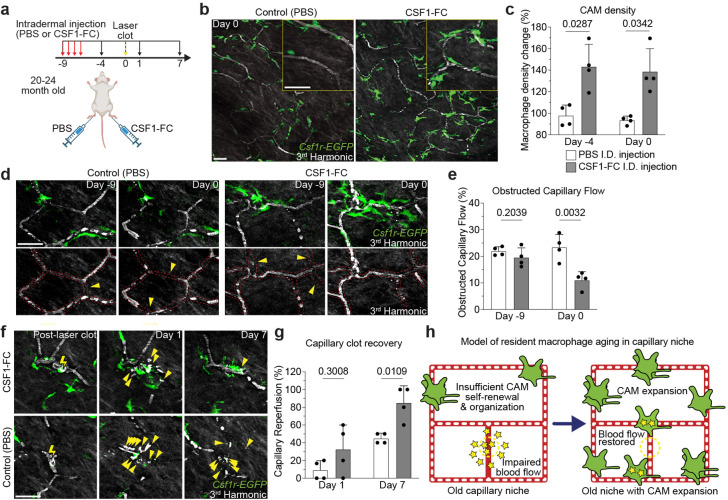
Local CAM replenishment in old mice is sufficient to rejuvenate capillary repair and tissue reperfusion. **a,** Scheme of CSF1-induced rejuvenation of aged capillary niche. **b,** Representative images of CAM density in *Csf1r-EGFP* mice nine days following daily intradermal injections (4 days) of CSF1-Fc (porcine CSF1 and IgG1a Fc region fusion protein) and PBS in contralateral hind paws of 20–24 month old mice. **c,** Quantification of CAM density following CSF1-Fc or PBS treatment (n = 4 mice in total; two 500μm^2^ regions of each treatment condition per mouse; Percentage of CAM density change from Day −9 (CSF1-Fc vs PBS) was compared for Day −4 and Day 0 time points by paired Student’s t test; mean ± SD). **d,** Representative images of capillary (red dashed lines) blood flow in *Csf1r-EGFP* mice nine days following daily intradermal injections of CSF1-Fc and PBS in contralateral hind paws of 20–24 month old mice. Yellow arrowheads indicate obstructed RBC capillary flow. **e,** Quantification of obstructed capillary blood flow following CSF1-Fc or PBS treatment (n = 214 capillary segments in CSF1-Fc treated regions, n = 214 capillary segments in PBS treated regions; n = 4 mice in total; obstructed capillary flow (CSF1-Fc vs PBS) was compared for Day −9 and Day 0 time points by paired Student’s t test; mean ± SD). **f,** Sequential revisits of damaged capillary niche after laser-induced clot formation (yellow lightning bolt) in *Csf1r-EGFP* mice. Yellow arrowheads indicate extra-luminal vascular debris. **g,** Quantification of capillary reperfusion at Day 1 and 7 after laser-induced clotting (n = 18 clots in CSF1-Fc group; n = 20 clots in PBS group; n = 4 mice in total; capillary reperfusion (CSF1-Fc vs PBS) was compared by paired Student’s t test; mean ± SD). **h,** Working model of resident macrophage aging in the skin capillary niche. Age-associated impairment in capillary repair can be rejuvenated following local expansion of resident macrophage population. Scale bar, 50μm.

Our results suggest that the capillary-associated macrophage population in old mice can be stably expanded following environmental changes, such as injury. Therefore, we next assessed if increased CAM density, without previous injury, would be sufficient to improve future capillary repair and reperfusion. To this end, we utilized a fusion protein of the canonical macrophage growth factor, CSF1, with the Fc region of porcine IgG, as it has been reported to robustly increase macrophage density in multiple tissues, including skin^30–32^. We performed daily intradermal injections of either CSF1-Fc or PBS in the left or right hind paws, respectively, of the same mice (Figure 4a). There was a significant increase in CAMs in the CSF1-treated paws compared to contralateral PBS controls, which had no significant change from pretreatment (Figure 4b,c). Strikingly, we found that CSF1 treatment was sufficient to improve homeostatic capillary blood flow in old mice, as compared to PBS controls which had significantly more obstructed capillary segments (Figure 4d,e). Utilizing the same aged mice, we next tested if this increase in CAM density would be sufficient to improve capillary repair rates. Following laser-induced clotting, there was a significant improvement in capillary repair and reperfusion in CSF1-treated mice compared to PBS controls (Figure 4 f,g). Consistent with this result, there was also significant improvement in capillary repair and tissue reperfusion in old mice following damage-induced CAM expansion (Extended Data Fig. 10i).

## Discussion

Macrophage renewal has largely been studied in non-physiological settings, such as through *in vitro* cell culture or severe depletion models that often are accompanied by acute inflammation^9,10,33–35^. Our work clearly demonstrates that homeostatic renewal of resident macrophages is not as finely tuned as has been previously suggested. Specifically, we found that neighboring macrophages do not proliferate or reorganize sufficiently to maintain an optimal population across the skin capillary niche unless they receive additional cues from acute tissue damage or increased growth factor abundance. Interestingly, we confirmed previous findings that show epidermal Langerhans cell density also declines with age, which has been associated with impaired epidermal function^36–39^. This raises the possibility that age-associated loss in macrophage density is a more general phenomenon in populations that rely on local self-renewal.

In addition to self-renewal, we also found that CAM recruitment to repair tissue damage was spatially restricted. To our knowledge, the long-term size and stability of resident macrophage territories or niches *in vivo* has not been reported. Our work provides strong evidence that injury-induced macrophage recruitment is restricted to approximately 100μm. In young mice, CAM density is high enough to provide substantial niche/territory overlap between neighbors. However, with declining CAM density with age, we show that a significant fraction of the skin capillary network is no longer within a CAM’s territory range. It will be important to understand how this property of CAMs is influenced by other aspects of regional heterogeneity, such as innate immune imprinting^13^, to shape local immune responses in tissues.

Collectively, this work demonstrates that loss in CAMs: 1) begins within the first few months of life, 2) is progressive throughout life, and 3) is functionally detrimental to vascular function, which has been shown to be a primary driver of age-associated tissue impairments^15,40^. Furthermore, this work provides a novel platform to investigate age-associated deviations in tissue homeostasis at the single-cell level in a living mammal.

## Methods

### Mice

Mice were bred and maintained in the Alexandria Center for the Life Sciences animal facility of the New York University School of Medicine, in specific pathogen-free conditions. Albino B6 (B6(Cg)-*Tyr*^*c-2J*^/J, Jax 000058), Csf1r^EGFP^ (B6.Cg-Tg(Csf1r-EGFP)1Hume/J, Jax 018549), Ccr2^RFP^ (B6.129(Cg)-Ccr2tm2.1Ifc/J, Jax 017586), R26^mTmG^ (B6.129(Cg)-Gt(ROSA)26Sortm4(ACTB-tdTomato,-EGFP)Luo/J, Jax 007676), R26^nTnG^ (B6N.129S6-Gt(ROSA)26Sortm1(CAG-tdTomato*,-EGFP*)Ees/J, Jax 023537), LysM^Cre^ (B6.129P2-Lyz2tm1(cre)Ifo/J, Jax 004781), Rac1^f/f^ (Rac1tm1Djk/J, Jax 005550) mice were purchased from Jackson Laboratories. *R26*^*dsRed*^ mice were described previously (Luche et al., 2007) and were obtained from the laboratory of Dr. Gordon Fishell. *Cx3cr1*^*CreER*^, *Cx3cr1*^*GFP*^*, and Cx3cr1*^*DTR*^ were generated in our laboratory and have been described (3 ref). Cre-induction for the lineage tracing or total CAM labeling experiments was induced with a single intraperitoneal injection of Tamoxifen (Sigma-Aldrich; T5648) (100μg or 4mg in corn oil, respectively) in 1 month old mice. *Rac1*^*f/f*^ recombination was induced with two intraperitoneal injections of Tamoxifen (2mg in corn oil) 48hrs apart in 1 month old mice. All imaging and experimental manipulation are performed on non-hairy mouse plantar (hind paw) skin. Preparation of skin for intravital imaging are performed as described below. Briefly, mice are anesthetized with intraperitoneal injection of ketamine/xylazine (15 mg/ml and 1 mg/ml, respectively in PBS). After imaging, mice are returned to their housing facility. For subsequent revisits, the same mice are processed again with injectable anesthesia. The plantar epidermal regions are briefly cleaned with PBS pH 7.2, mounted on a custom-made stage and a glass coverslip are placed directly against the skin. Anesthesia is maintained throughout the course of the experiment with vaporized isoflurane delivered by a nose cone. Mice from experimental and control groups were randomly selected for live imaging experiments. No blinding was done. All lineage tracing and ablation experiments were repeated in at least three different mice. All animal procedures were performed in accordance with protocols approved by the Institutional Animal Care and Usage Committee of New York University School of Medicine.

### *In vivo* imaging and laser ablation

Image stacks were acquired with an Olympus multiphoton FVMPE-RS system equipped with both InSight X3 and Mai Tai Deepsee (Spectra-Physics) tunable Ti:Sapphire lasers, using Fluoview software. For collection of serial optical sections, a laser beam (940nm for GFP/tdTomato/dsRed/RFP/Rhodamine/Second Harmonic Generation and 1300nm for Third Harmonic Generation, respectively) was focused through a water immersion lens (N.A. 1.05; Olympus) and scanned with a field of view of 0.5mm^2^, at 600 Hz. Z-stacks were acquired in 1–2μm steps for a ~50–100μm range, covering the epidermis and dermis. Capillary blood flow was visualized in some experiments through intravenous injection with 18 mg/kg of dextran-rhodamine 70 kD (Sigma-Aldrich; R9379). Cell tracking analysis was performed by re-visiting the same area of the dermis in separate imaging experiments through using inherent landmarks of the skin to navigate back to the original region, including the distinct organization of the superficial vasculature networks. Cells that were unambiguously separated (by at least 250μm) from another were sampled to ensure the identity of individual lineages. For time-lapse recordings, serial optical sections were obtained between 5–10 minute intervals, depending on the experimental setup. Laser-induced cell ablation, capillary clot, or tissue damage was carried out with the same optics as used for acquisition. An 940nm laser beam was used to scan the target area (1–500μm^2^) and ablation was achieved using 50–70% laser power for ~1sec. Ablation parameters were adjusted according to the depth of the target (10–50μm). Mice from experimental and control groups were randomly selected for live imaging experiments. No blinding was done. All lineage tracing and ablation experiments were repeated in at least three different mice.

### Drug treatments

To induce macrophage depletion, mice received intradermal injections of either Clodronate-liposomes or PBS-liposomes (stock concentration 5mg/ml; Liposoma; CP-005–005) (5μl per paw) every 3 days. Depending on experimental details, Cx3cr1^DTR^ mice received either intraperitoneal (IP) injection of diphtheria toxin (Sigma-Aldrich; D0564) every other day at 25ng/g body weight in PBS or a single low dose IP injection at 10ng/g body weight in PBS. To induce macrophage expansion, mice receive daily intradermal injections of CSF1-FC (Bio-Rad; PPP031) or PBS in contralateral hind paws (5μl per paw) for 4 days.

### Human skin samples

Written informed consent was obtained for postmortem examination from next of kin for 10 patients. Clinical information and laboratory data were obtained from the electronic medical record. Skin samples were obtained from the anterolateral chest and fixed in 10% formalin for at least 24h prior to processing. Slides were stained with hematoxylin and eosin, CD68 (Clone 514H12) and ERG (Clone EPR3864). Macrophages and capillaries were identified using a combination of morphology, CD68 and ERG staining. Counting was performed on at least eight high-power fields (40x) within 100um of the epidermis.

### Image Analysis

Raw image stacks were imported into Fiji (NIH, USA) or Imaris software (Bitplane/Perkin Elmer) for further analysis. Provided images and supplementary videos are typically presented as a maximal projection of 4–8μm optical sections. For visualizing individual labeled cells expressing the dsRed or tdTomato Cre reporters, the brightness and contrast were adjusted accordingly for the green (GFP) and red (dsRed/tdTomato) channels and composite serial image sequences were assembled as previously described. Images were obtained as large tiled image stacks at roughly the same positions and then manually aligned over the experimental time course in Imaris (Bitplane/Perkin Elmer) by using data from all channels.

### Statistical Analysis

Data are expressed as mean ± SD. An unpaired Student’s *t*-test was used to analyze data sets with two groups. One-way ANOVA was used to analyze data sets with three or more groups. *p* < 0.05 to *p* < 0.0001 indicated a significant difference. Statistical calculations were performed using the Prism software package (GraphPad, USA).

## Extended Data

## Supplementary Material

Supplement 1 **Supplemental Video 1.** Serial optical sections through mouse plantar skin, including epidermis (0–25μm), upper dermis (26–50μm), and lower dermis (51–100μm) in *R26-mTmG* (red) mice. Note Second and Third Harmonic Generation illuminates dermal collagen (blue) and red blood cells (white), respectively. Scale bar, 50μm.

Supplement 2 **Supplemental Video 2.** Serial optical sections through mouse plantar skin, including epidermis (0–15μm), upper dermis (16–40μm), and lower dermis (41–52μm) in *Csf1-rEGFP* mice. Note distinct macrophage (green) density and morphology in each tissue niche. Scale bar, 50μm.

Supplement 3 **Supplemental Video 3.** Time-lapse recording of capillary blood flow via third harmonic generation (white) from red blood cells and intravenous rhodamine dextran (red) in *Csf1r-EGFP* mice. Note some capillary segments show obstructed red blood cell and rhodamine dextran flow. Scale bar, 50μm.

Supplement 4 **Supplemental Video 4.** Serial optical sections through upper dermal capillary niche in *Cx3cr1-GFP; R26-mTmG* mice. Note macrophage (green) localization and morphology around capillary (red) network. Scale bar, 50μm.

Supplement 5 **Supplemental Video 5.** Time-lapse recording of capillary blood flow in *Cx3cr1-CreER; R26-mTmG* mice. Note inconsistent and obstructed blood flow (white) in capillary (red) segments without associated macrophages (green). Scale bar, 50μm.

Supplement 6 **Supplemental Video 6.** Time-lapse recording following laser-induced capillary clot formation in *Csf1r-GFP; R26-mTmG* mice. Note rapid migration of neighboring capillary-associated macrophages (green) toward site of clot formation (yellow arrowhead). Scale bar, 50μm.

## References

[1] Nobs S. P. & Kopf M. Tissue-resident macrophages: guardians of organ homeostasis. Trends Immunol 42, 495–507 (2021). 33972166 10.1016/j.it.2021.04.007

[2] Minutti C. M., Knipper J. A., Allen J. E. & Zaiss D. Tissue-specific contribution of macrophages to wound healing. Semin Cell Dev Biol 61, 3–11 (2017). 27521521 10.1016/j.semcdb.2016.08.006

[3] Vannella K. M. & Wynn T. A. Mechanisms of Organ Injury and Repair by Macrophages. Annual Review of Physiology 79, (2016). 10.1146/annurev-physiol-022516-034356 27959618

[4] Chakarov S. Two distinct interstitial macrophage populations coexist across tissues in specific subtissular niches. Science 363, eaau0964 (2019). 30872492 10.1126/science.aau0964

[5] Wu Y. & Hirschi K. K. Tissue-Resident Macrophage Development and Function. Frontiers Cell Dev Biology 8, 617879 (2021). 10.3389/fcell.2020.617879 PMC7820365 33490082

[6] Beek A. A. van, Bossche, J. den, Mastroberardino, P. G., Winther, M. de & Leenen, P. Metabolic Alterations in Aging Macrophages: Ingredients for Inflammaging? Trends Immunol (2019) doi:10.1016/j.it.2018.12.007. 30626541

[7] Franceschi C., Garagnani P., Vitale G., Capri M. & Salvioli S. Inflammaging and ‘Garb-aging.’ Trends Endocrinol Metabolism 28, 199–212 (2017). 10.1016/j.tem.2016.09.005 27789101

[8] Mass E., Nimmerjahn F., Kierdorf K. & Schlitzer A. Tissue-specific macrophages: how they develop and choreograph tissue biology. Nat. Rev. Immunol. 1–17 (2023) doi:10.1038/s41577-023-00848-y. PMC10017071 36922638

[9] Bruttger J. Genetic Cell Ablation Reveals Clusters of Local Self-Renewing Microglia in the Mammalian Central Nervous System. Immunity 43, 92–106 (2015). 26163371 10.1016/j.immuni.2015.06.012

[10] Sakai M. Liver-Derived Signals Sequentially Reprogram Myeloid Enhancers to Initiate and Maintain Kupffer Cell Identity. Immunity 51, 655–670.e8 (2019). 31587991 10.1016/j.immuni.2019.09.002 PMC6800814

[11] Hashimoto D. Tissue-Resident Macrophages Self-Maintain Locally throughout Adult Life with Minimal Contribution from Circulating Monocytes. Immunity 38, 792–804 (2013). 23601688 10.1016/j.immuni.2013.04.004 PMC3853406

[12] Okabe Y. & Medzhitov R. Tissue biology perspective on macrophages. Nature Immunology 17, 9–17 (2016). 26681457 10.1038/ni.3320

[13] Guilliams M., Thierry G. R., Bonnardel J. & Bajenoff M. Establishment and Maintenance of the Macrophage Niche. Immunity 52, 434–451 (2020). 32187515 10.1016/j.immuni.2020.02.015

[14] Fukada K. & Kajiya K. Age-related structural alterations of skeletal muscles and associated capillaries. Angiogenesis 23, 79–82 (2020). 31993832 10.1007/s10456-020-09705-1

[15] Grunewald M. Counteracting age-related VEGF signaling insufficiency promotes healthy aging and extends life span. Science 373, eabc8479 (2021). 34326210 10.1126/science.abc8479

[16] Pluvinage J. V. & Wyss-Coray T. Systemic factors as mediators of brain homeostasis, ageing and neurodegeneration. Nat Rev Neurosci 21, 93–102 (2020). 31913356 10.1038/s41583-019-0255-9

[17] Shaw A. C., Goldstein D. R. & Montgomery R. R. Age-dependent dysregulation of innate immunity. Nature reviews. Immunology (2013) doi:10.1038/nri3547. PMC4096436 24157572

[18] Pineda C. M. Intravital imaging of hair follicle regeneration in the mouse. Nature protocols 10, 1116–30 (2015). 26110716 10.1038/nprot.2015.070 PMC4632978

[19] Mesa K. R. Homeostatic Epidermal Stem Cell Self-Renewal Is Driven by Local Differentiation. Cell Stem Cell (2018) doi:10.1016/j.stem.2018.09.005. PMC6214709 30269903

[20] Dietzel S. Label-Free Determination of Hemodynamic Parameters in the Microcirculaton with Third Harmonic Generation Microscopy. PLoS ONE 9, e99615 (2014). 24933027 10.1371/journal.pone.0099615 PMC4059650

[21] Saytashev I. Multiphoton excited hemoglobin fluorescence and third harmonic generation for non-invasive microscopy of stored blood. Biomedical Optics Express 7, 3449 (2016). 27699111 10.1364/BOE.7.003449 PMC5030023

[22] Bentov I. & Reed M. J. The effect of aging on the cutaneous microvasculature. Microvasc Res 100, 25–31 (2015). 25917013 10.1016/j.mvr.2015.04.004 PMC4461519

[23] Smith L. Histopathologic characteristics and ultrastructure of aging skin. Cutis 43, 414–24 (1989). 2721240

[24] Li L. Age-Related Changes of the Cutaneous Microcirculation in vivo. Gerontology 52, 142–153 (2006). 16645294 10.1159/000091823

[25] Reeson P., Choi K. & Brown C. E. VEGF signaling regulates the fate of obstructed capillaries in mouse cortex. eLife 7, e33670 (2018). 29697373 10.7554/eLife.33670 PMC5919759

[26] Das A. Impairment of an Endothelial NAD+-H2S Signaling Network Is a Reversible Cause of Vascular Aging. Cell 173, 74–89.e20 (2018). 29570999 10.1016/j.cell.2018.02.008 PMC5884172

[27] Cox D. Requirements for Both Rac1 and Cdc42 in Membrane Ruffling and Phagocytosis in Leukocytes. J Exp Medicine 186, 1487–1494 (1997). 10.1084/jem.186.9.1487 PMC2199122 9348306

[28] Perdiguero E. & Geissmann F. The development and maintenance of resident macrophages. Nat Immunol 17, 2–8 (2015). 10.1038/ni.3341 PMC4950995 26681456

[29] Blériot C., Chakarov S. & Ginhoux F. Determinants of Resident Tissue Macrophage Identity and Function. Immunity 52, 957–970 (2020). 32553181 10.1016/j.immuni.2020.05.014

[30] Alfituri O. A., Mararo E. M., Steketee P. C., Morrison L. J. & Mabbott N. A. Dermal bacterial LPS-stimulation reduces susceptibility to intradermal Trypanosoma brucei infection. Sci Rep-uk 11, 9856 (2021). 10.1038/s41598-021-89053-2 PMC8110744 33972588

[31] Gow D. J. Characterisation of a Novel Fc Conjugate of Macrophage Colony-stimulating Factor. Mol Ther 22, 1580–1592 (2014). 24962162 10.1038/mt.2014.112 PMC4435485

[32] Keshvari S. Therapeutic potential of macrophage colony-stimulating factor in chronic liver disease. Dis Model Mech 15, dmm049387 (2022). 35169835 10.1242/dmm.049387 PMC9044210

[33] Zhou X. Circuit Design Features of a Stable Two-Cell System. Cell 172, 744–757.e17 (2018). 29398113 10.1016/j.cell.2018.01.015 PMC7377352

[34] Nicolás-Ávila J. A. A Network of Macrophages Supports Mitochondrial Homeostasis in the Heart. Cell 183, 94–109.e23 (2020). 32937105 10.1016/j.cell.2020.08.031

[35] Ferrer I. R. A wave of monocytes is recruited to replenish the long-term Langerhans cell network after immune injury. Sci Immunol 4, eaax8704 (2019). 31444235 10.1126/sciimmunol.aax8704 PMC6894529

[36] Gilchrest B. A., Murphy G. F. & Soter N. A. Effect of Chronologic Aging and Ultraviolet Irradiation on Langerhans Cells in Human Epidermis. J Invest Dermatol 79, 85–88 (1982). 7097040 10.1111/1523-1747.ep12500031

[37] Cruchley A. T. Langerhans cell density in normal human oral mucosa and skin: relationship to age, smoking and alcohol consumption. J Oral Pathol Med 23, 55–59 (1994). 8164153 10.1111/j.1600-0714.1994.tb00256.x

[38] Hasegawa T. Reduction in human epidermal Langerhans cells with age is associated with decline in CXCL14-mediated recruitment of CD14+ monocytes. J Invest Dermatol (2019) doi:10.1016/j.jid.2019.11.017. PMC8142052 31881212

[39] Fenske N. A. & Lober C. W. Structural and functional changes of normal aging skin. J Am Acad Dermatol 15, 571–585 (1986). 3534008 10.1016/s0190-9622(86)70208-9

[40] Cai C. Impaired dynamics of precapillary sphincters and pericytes at first-order capillaries predict reduced neurovascular function in the aging mouse brain. Nat Aging 3, 173–184 (2023). 37118115 10.1038/s43587-022-00354-1 PMC11081516

